# Power and sample size considerations for test-negative design with bias correction: a case study on the world first malaria vaccine

**DOI:** 10.1186/s12874-025-02628-9

**Published:** 2025-07-29

**Authors:** Yura K. Ko, Tobias Alfvén, Daisuke Yoneoka

**Affiliations:** 1 Department of Microbiology, Tumor and Cell Biology (MTC), Karolinska Institutet, Stockholm, Sweden; 2 Department of Virology, Tohoku University Graduate School of Medicine, Sendai, Japan; 3 Department of Global Public Health, Karolinska Institutet, Stockholm, Sweden; 4 Sachs’ Children and Youth Hospital, Stockholm, Sweden; 5 Center for Surveillance, Immunization, and Epidemiologic Research, National Institute of Infectious Diseases, Tokyo, Japan

**Keywords:** Test negative design, Bias correction, Sample size, Statistical power, Vaccine effectiveness, Malaria

## Abstract

**Background:**

Test-negative design (TND) studies are increasingly common in evaluating vaccine effectiveness (VE) for various infectious diseases. TND studies are susceptible to bias due to disease outcome misclassification caused by imperfect test sensitivity and specificity. Several bias correction methods have been proposed. However, sample size or power considerations for TND studies incorporating bias correction for such misclassification have not yet been investigated.

**Methods:**

We used Monte Carlo simulations to assess how bias correction affects the statistical power and sample size for VE estimation in TND studies. Simulations were conducted under varying levels of diagnostic test sensitivities (60%, 80%, and 95%). Bias correction was implemented using the multiple over-imputation method, which accounts for test misclassification through a parametric bootstrapping approach. Using a malaria vaccine as an example, we defined six vaccination status categories based on the time since receipt of the third or fourth vaccine dose. In the simulated target population, vaccination coverage was assumed to be low (< 10%) except for the group vaccinated more than 12 months after dose 4. We assumed relatively low VE (< 50%) against clinical malaria cases and a 30% malaria positivity rate among unvaccinated individuals presenting with malaria-related symptoms. Statistical power to detect VE was estimated for each vaccination status, both with and without bias correction.

**Results:**

Estimated VEs based on observed data were consistently underestimated across all vaccination status groups due to diagnostic misclassification. In contrast, bias-corrected estimates were approximately unbiased but displayed wider confidence intervals, with their precision decreasing at lower test sensitivities. Statistical power to detect VE declined substantially when diagnostic test sensitivity was low. For instance, at 80% sensitivity, only three vaccination status groups reached 80% power with a sample size of 10,000, whereas the same power was achieved with just 6,000 individuals under a perfect test.

**Conclusions:**

Bias due to imperfect diagnostic testing can substantially reduce the power of TND studies. Power calculations should account for outcome misclassification and potential correction methods. Failure to do so may lead to underpowered studies and misleading VE estimates.

**Supplementary Information:**

The online version contains supplementary material available at 10.1186/s12874-025-02628-9.

## Background

Test-negative design (TND) studies are increasingly common in evaluating vaccine effectiveness (VE) for various infectious diseases [1]. Historically, the design has been applied to influenza vaccines [2] and since the pandemic in 2020, it has been adopted in many countries, including those in Africa [3–5] to estimate the VE of COVID-19 vaccines due to its efficiency. More recently, TND has been employed to evaluate novel vaccines for respiratory syncytial virus (RSV) [6] started in 2023.

In a TND study, cases and controls are enrolled from the same location using the same clinical case definitions. This approach minimizes potential selection bias associated with health-seeking behavior compared to traditional case-control studies [1]. However, one of the limitations of TND is its vulnerability to misclassification of disease outcomes caused by the imperfect sensitivity and specificity of diagnostic tests [7]. Jackson et al. conducted a simulation study showing that influenza test misclassification led to greater VE underestimation in TND than in traditional cohort or case-control studies [7].

To address this, Endo et al. proposed a bias correction method [8] that can be applied using existing statistical software for logistic regression, the most common approach for estimating VE. They demonstrated that the correction method yields unbiased VE estimates, albeit with wider confidence intervals. Consequently, when less sensitive and/or specific tests, such as rapid diagnostic tests (RDTs), are used, a larger sample size is required to estimate unbiased VE with sufficient statistical power. However, sample size or power considerations for TND studies incorporating bias correction have not yet been investigated.

In this study, we explore how bias correction influences statistical power, using the malaria vaccine RTS, S/AS01, as a motivating example. We employ Monte Carlo simulations because they offer a highly flexible framework for exploring a wide range of scenarios and can be readily expanded when new data or assumptions arise in future studies, including mixed/random-effect modeling for heterogenetiy between individuals. There are two primary reasons for focusing on the malaria vaccine. First, RTS, S/AS01 is the world’s first malaria vaccine, recommended by the World Health Organization (WHO) in 2021 and prequalified in 2022. To date, no studies have evaluated the real-world VE of RTS, S/AS01 using TND. Second, in most clinical settings, the most common diagnostic tool for malaria is RDTs, which are highly specific but have relatively lower sensitivity [9, 10]. We used VE estimates from a phase-3 trial conducted in seven sub-Saharan African countries [11] and a subsequent mathematical model study [12] for our simulation.

This study aims to provide field epidemiologists, particularly those in African countries, with practical guidance for designing their first TND studies in such contexts. To facilitate this, we present a Shiny application that enables users to estimate VE by customizing their assumptions.

## Methods

We conducted Monte-Carlo simulations to estimate VE with and without bias correction and statistical power given different sample sizes (*N* = 5000, 6000, 7000, 8000, 9000, 10,000). The total number of simulated datasets was set to 1,000 iterations to balance the reduction of simulation error with the feasibility of completing the simulation study within a reasonable time. All simulations and estimations were performed using Julia software (version 1.10.3). Reproducible code is available on GitHub (https://github.com/KoKYura/TND_power). Additionally, a Shiny application is available to calculate power by changing parameters and sample size (See the Supplementary file for detailed instructions on using the application).

### Simulation settings

We considered $$\:S$$ mutually exclusive vaccination status categories. Let $$\:{\varvec{p}}_{\varvec{s}}\in\:{R}^{s}$$ denote the vector of proportions of individuals in each vaccination status category among participants. For each individual $$\:\varvec{i}$$, the vaccination status vector $$\:{\varvec{x}}_{\varvec{i}}$$$$\:=\left({x}_{1i},\dots\:,\:{x}_{Si}\right)$$ was drawn from a categorical distribution with probabilities $$\:{\varvec{p}}_{\varvec{s}}$$, such that $$\:{x}_{si}=\left\{0,\:1\right\}$$ and $$\:{\sum\:}_{s=1}^{S}{x}_{si}=1$$. The true malaria infection outcome $$\:{y}_{i}^{true}$$ was then generated as:

$$\:y_i^{true}\:\sim\:Bernoulli\left(\text{e}\text{x}\text{p}\text{i}\text{t}\left(\text{l}\text{o}\text{g}\text{i}\text{t}\left(\text{P}\left(TD\right)\right)+\:{\textstyle\sum_s}\;x_{si}\cdot\:\text{l}\text{o}\text{g}\left(1-{VE}_s\right)\right)\right),\:$$

where $\:logit\left(x\right)=\text{l}\text{o}\text{g}\left(\frac{x}{1-x}\right)$, $\:expit\left(x\right)=\frac{1}{1+\text{e}\text{x}\text{p}(-x)}$. Here, $\:P\left(T{D}_{\:}\right)$ represents the probability of being diagnosed with the target disease (TD) upon showing symptoms without vaccination, and $\:{VE}_{s}\in\:[0,\:1)$ denotes the vaccine effectiveness against clinical illness for vaccination status $\:s$. The number of vaccination status can be unlimited (e.g., more than x days after vaccination, within x days after vaccination, and not vaccinated). However, as the categories being more detailed, the proportion for each status decreases, which in turn reduces statistical power. As a key assumption in TND studies, we assumed that vaccines have no effect on the risk of non-target diseases (ND). Therefore, the proportion of each vaccination status among individuals with ND ($\:{y}_{i}^{true}\:$ = 0) can be considered the same as the proportion of each vaccination status in the general population, which is obtainable data defined as $\:{\varvec{p}\varvec{{\prime\:}}}_{\varvec{s}}$. Then, using the ratio of TD to ND for each vaccination status, $\:P\left(T{D}_{s}\right)/P\left(N{D}_{s}\right)$, we get the estimates of $\:{\varvec{p}}_{\varvec{s}}$ as $\:{p{\prime\:}}_{s}(1+P(T{D}_{s})/P(N{D}_{s}\left)\right)$. Lastly, we defined sensitivity (sen) and specificity (spe) as $\:sen=\:P\left({y}_{i}^{obs}=1|{y}_{i}^{true}=1\right)$ and $\:spe=\:P\left({y}_{i}^{obs}=0\:|{y}_{i}^{true}=0\right)$, respectively. Then, the observed $\:{y}_{i}^{obs}$ was randomly sampled from Bernoulli distribution as follows:

$$\:{y}_{i}^{obs}\sim\:\left\{\begin{array}{c}Bernoulli\left(sen\right)\:\:\:\:\:\:if\:{y}_{i}^{true}=1,\:\:\\\:Bernoulli\left(1-spe\right)\:\:\:\:\:\:if\:{y}_{i}^{true}=0.\end{array}\right.$$

### RTS, S/AS01 malaria vaccine

The following assumptions were made to design a TND study for estimating the VE of the malaria vaccine RTS, S/AS01. The vaccine has a four-dose regimen with a three-dose primary series given at a minimum interval of four weeks between doses in children from five months of age, followed by a booster dose 12–18 months after the third dose. In the phase-3 trial, the vaccine showed modest efficacy against *Plasmodium falciparum* malaria [11]. A subsequent mathematical modeling analysis estimated the time-specific efficacy against clinical disease for the periods after the third dose, both with and without the booster vaccination [12]. Using those estimates, we specified the expected VE against clinical malaria for each post-dose interval (Table 1). Because of the vaccination schedule and its relatively short duration of protection [11], the target population was defined as children aged two to five years. For this age range, we assumed the vaccination coverage ($\:{\varvec{p}\varvec{{\prime\:}}}_{\varvec{s}}$) listed in Table 1, based on the previous study in Kenya; Kenya is one of the three countries that began RTS, S/AS01 pilot implementation in 2019 [13]. We employed malaria RDT sensitivities of 60%, 80%, and 95%, and a specificity of 98%, based on several studies [9, 10]. The probability of malaria positivity among individuals presenting with malaria-related symptoms without vaccine history ($\:P\left(T{D}_{\:}\right)$) was assumed to be 30%, based on an ongoing cohort study (unpublished data) [14] in Homa Bay County, Kenya.

**Table 1:** The assumption of general proportion among children aged 2–5 years old and VE against clinical malaria of each vaccination status

Vaccination status	Proportion among general population	VE against clinical malaria
**Without dose 4**		
24 – months, Dose 3 or without Dose 3	32.8%	0% (as reference)
–6 months, Dose 3	2.6%	50%
6–12 months, Dose 3	2.6%	30%
12–24 months, Dose 3	4.5%	10%
**With dose 4**		
–6 months, Dose 4	6.3%	50%
6–12 months, Dose 4	6.3%	30%
12– months, Dose 4	44.9%	10%

### VE estimation with bias correction

Following Endo et al. [8] we generated M = 100 pseudo-complete data sets by probabilistically ‘flipping’ each observed test result according to pre-specified or externally estimated sensitivity (sen) and specificity (spe). For an individual with $\:{y}_{i}^{obs}=1$, the imputed true outcome $\:{y}_{i}^{*\left(m\right)}$ in the *m*th data set was set to 1 with probability sen and to 0 with probability 1-sen. Conversely, for those with $\:{y}_{i}^{obs}=0$, $\:{y}_{i}^{*\left(m\right)}$ was set to 1 with probability 1-spe and to 0 with probability spe. Each imputed data set was then analyzed with the same logistic regression model used for the main analysis, estimating the Odds ratio (OR). Lastly, the estimated OR in each dataset was combined across M imputations using Rubin’s rules, thereby accounting for both within- and between-imputation variability. The VE for each vaccination status was then estimated, with 95% Confidence Intervals (CI) calculated based on 2.5th and 97.5th percentiles of the estimates from the bootstrapped datasets. The detailed methodology is described elsewhere [8].

### Power calculation and alpha error

Each statistical power for detecting true, observed, and bias-corrected VEs was defined as the proportion of datasets for which the 95% CI for the corresponding VE does not include Null value (i.e., 0).

Data sets were also simulated under the assumption of null VEs for all vaccination statuses to evaluate the probability of reporting a spurious VE where the vaccine has no effect. The alpha error was defined as the proportion of datasets under the null hypothesis for which the 95% CI for the corresponding VE includes 0.

## Results

### The alpha error for spurious estimation of VE

The alpha error for each sample size, when bias correction was conducted for an imperfect diagnostic test with sensitivities of 80%, 60%, and 95%, is presented in Table 2, Supplementary Table 1, and Supplementary Table 2, respectively. The results demonstrated that all simulations yielded an alpha error of less than 5%.

**Table 2:** The alpha error when the bias correction conducted for an imperfect test with a sensitivity of 80% and a specificity of 98% in each sample size for each vaccination status

*N*	–6 months, Dose 3	6–12 months, Dose 3	12–24 months, Dose 3	–6 months, Dose 4	6–12 months, Dose 4	12– months, Dose 4
5000	0.035	0.034	0.012	0.020	0.022	0.019
6000	0.032	0.026	0.016	0.018	0.028	0.015
7000	0.030	0.024	0.021	0.025	0.024	0.023
8000	0.022	0.024	0.028	0.027	0.028	0.020
9000	0.030	0.024	0.026	0.019	0.022	0.021
10,000	0.024	0.030	0.025	0.030	0.021	0.025

### Accuracy and precision of the estimated VEs

**Fig. 1:**
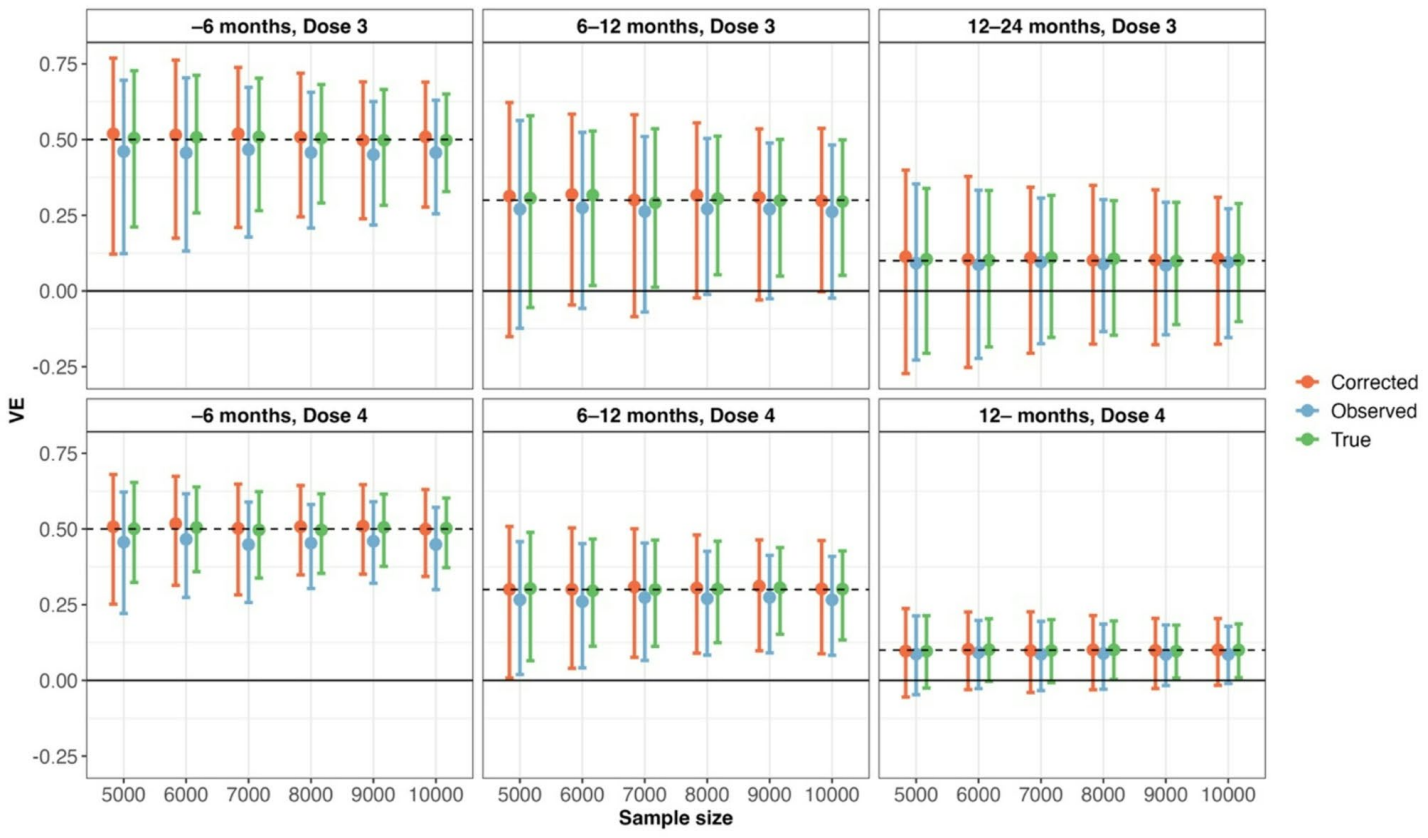
Estimated true, observed, and bias-corrected vaccine effectiveness (VE) under an imperfect diagnostic test with 80% sensitivity and 98% specificity. Estimates are presented with 95% coverage intervals based on 1,000 Monte Carlo simulations, stratified by vaccination status group and sample size. The figure illustrates the degree of bias in VE estimates due to diagnostic misclassification and the extent to which bias correction restores validity

Figure 1, Supplementary Fig. 1, and Supplementary Fig. 2 show the estimated VEs with 95% CI across 1,000 simulations. In all vaccination status groups, the estimated VEs based on observed data were consistently underestimated. In contrast, the estimated VEs obtained using bias correction were unbiased but exhibited a wider range of estimates, which varied depending on the level of test sensitivity.

### Statistical power

**Fig. 2:**
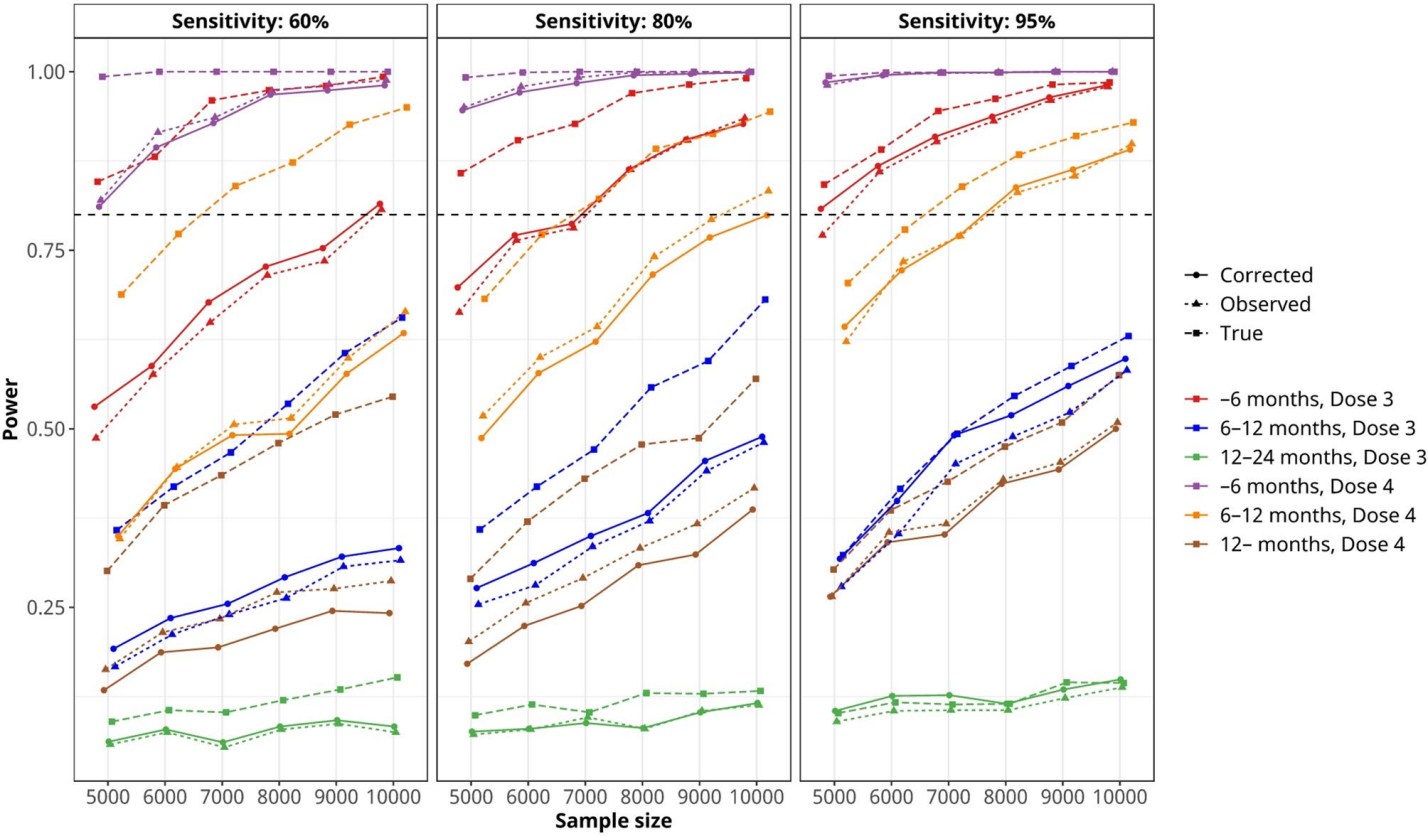
Estimated statistical power to detect vaccine effectiveness (VE) for true, observed, and bias-corrected estimates under imperfect diagnostic tests with sensitivities of 60%, 80%, and 95%, and a specificity of 98%. Results are based on 1,000 Monte Carlo simulations for each vaccination status group and sample size. The figure illustrates how test sensitivity and bias correction impact the power of VE estimation in simulation-based evaluations

Figure 2 shows the estimated statistical power for each vaccination status and sample size. When estimating VE using bias correction for an imperfect test with a sensitivity of 60% or 80% and a specificity of 98%, the power was substantially reduced compared to the power estimated using a perfect test. The power was nearly equivalent to that of bias-uncorrected estimates for imperfect tests. For example, with a sensitivity of 80%, only three vaccination status groups (–6 months post Dose 3, − 6 months post Dose 4, and 6–12 months post Dose 4) reached 80% power with a sample size of 10,000, while when using a perfect test, the power approached to 80% when the sample size was 6000 for these three groups. For highly sensitive tests (95% sensitivity), all three estimated VEs (true, observed, and bias-corrected) exhibited trivial differences in statistical power (the right panel of Fig. 2).

### Shiny application

**Fig. 3:**
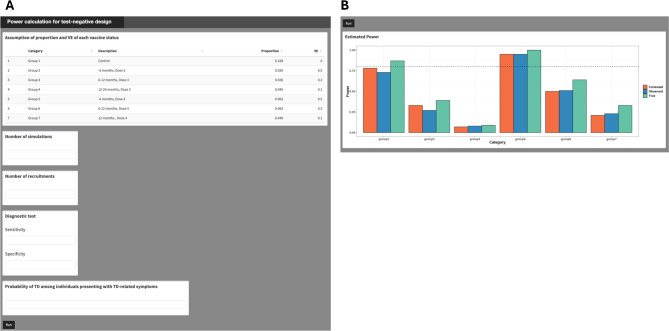
User interface **A** and output **B** of the Shiny application. By specifying the following assumptions—proportion and vaccine effectiveness (VE) for each vaccination status, number of simulations, total number of sample size, diagnostic test sensitivity and specificity, probability of being diagnosed as target disease (TD), the user can obtain the statistical power for each vaccination status

Figure 3 displays the input panel and output of the Shiny application. By specifying (1) the proportion of participants in each vaccination category and its expected VE; 2) the number of Monte Carlo simulations; 3) the total sample size; 4) the diagnostic test’s sensitivity and specificity; and 5) the probability of being diagnosed as TD, the user can calculate the statistical power for true, observed, and bias-corrected VEs of each vaccination group compared to the control group (Group 1 in Fig. 3A).

## Discussion

Our simulations demonstrated that using imperfect diagnostic tests reduces statistical power in both observed data-based VE and bias-corrected VE. The magnitude of power loss highly depends on the sensitivity of the tests. Given the limited research on power and sample size calculations for the TND [15], our study provides valuable insights for assessing study feasibility, particularly for infectious diseases that are regularly diagnosed with less sensitive tests. This includes considerations of target populations, the number of study sites, and the duration of participant recruitment.

The bias in VE estimation due to the misclassification of outcomes in TND studies has been recognized for a long time [7, 16, 17] and several bias correction methods have been proposed [8, 18]. However, there are very few reports of VE estimates after correcting bias using these methods. For example, among the studies citing the work of Endo et al., only Amin et al. applied bias correction for sensitivity and specificity using the proposed method [19] while Yoon et al. merely mentioned the bias as a study limitation [20]. Incorporating bias correction into the power calculation stage of research design could facilitate the wider adoption of bias-corrected VE estimation in future studies.

In this study, we used the world’s first malaria vaccine as a motivating example to simulate power for TND studies incorporating bias correction. Our simulation methods can also be applied to vaccines for other infectious diseases; however, several points should be addressed. First, our results indicated that higher VE corresponded to higher power. However, as shown in Supplementary Fig. 3, if VE is very high— such as above 90%, which is unlikely for malaria vaccines—the power may decline due to the limited number of vaccinated test-positive cases. In such scenarios, Huo et al. recommended using a score-based approach to design and analyze TND [15]. Second, we conducted simulations varying sensitivities (60%, 80%, 95%) while fixing the specificity at 98%. This decision was based on the fact that the specificity of RDTs is generally high, at 98% or above, including for malaria. Nevertheless, it should be noted that tests with lower specificity can have a greater impact on power [17]. Furthermore, when the probability of target diseases, P(TD), is considerably lower than that assumed for malaria, power also declines markedly. Using the same simulation framework, we evaluated both scenarios—reduced specificity and reduced P(TD)— and found that power decreased for all estimates (true, observed, and bias-corrected), as shown in Supplementary Fig. 4.

This study has several limitations. First, we conducted a univariate analysis, incorporating vaccination status as the sole explanatory variable, rather than conducting a multivariate analysis. It should be noted that including additional variables as confounders would reduce the expected power [8] depending on their effect size on the outcome and their (joint) distribution. Incorporating such assumptions into the simulation would require substantial prior information about the confounders, which is not feasible; therefore, we did not consider it in this study. Notably, the sample size calculation for evaluating the COVID-19 vaccine in TND, as published by the WHO, also assumes a univariate analysis [21]. Second, we considered a simplified scenario with constant vaccine coverage, no seasonal variation in TD risk, and, importantly, no change in infection prevention or risky behavior based on vaccination status. Although there is no clear evidence yet, if children vaccinated with the RTS, S/AS01 use mosquito nets less frequently in real-world settings, the effectiveness of the vaccine may be underestimated. In actual TND studies, it is important to conduct thorough interviews to assess these risky and preventive behaviors simultaneously. Third, we did not consider misclassification of vaccine status, which represents a potential source of bias in TND studies [22]. In the case of most childhood vaccines, vaccination records are maintained in the Mother-Child handbook. Ensuring that investigators verify these records, rather than relying solely on verbal responses, can minimize such misclassification. Finally, since our CIs are built from only 𝑀=100 parametric-bootstrap resamples, their actual coverage probability can fall short of the nominal level in small or highly misclassified samples even if the sample size increases (Table 2). Evaluating larger 𝑀 and alternative interval constructions (e.g., studentised or BCa) to recover nominal coverage is left for future work.

## Conclusions

In summary, we showed the simulated power for estimating bias-corrected VE when diagnostic tests have lower sensitivity using the malaria vaccine as a motivating example. During the design phase of a TND study, researchers should conduct power calculations accounting for correcting the bias due to outcome misclassification. To achieve this, researchers need to collect comprehensive data, including the expected effect size of VE, the sensitivity and specificity of the diagnostic tests, the proportion of the vaccinated group, and the case ratio of TD to ND. Such data can be obtained from pilot studies, published and/or unpublished data from the same region, and existing literature.

## Supplementary Information

Supplementary Material 1.

## Data Availability

Replication codes are available on GitHub (https://github.com/KoKYura/TND_power).
