# Supplementary material for: Power and sample size considerations for test-negative design with bias correction: a case study on the world first malaria vaccine
Source: BMC Med Res Methodol. 2025 Jul 29;25:178. doi: 10.1186/s12874-025-02628-9 (PMC12309064; doi:10.1186/s12874-025-02628-9)
Supplement: Supplementary file 1 — Supplementary Material 1. [file 12874_2025_2628_MOESM1_ESM.docx]

**Power and sample size considerations for test-negative design with bias correction: a case study on the world first malaria vaccine**

**Authors:** Yura K. Ko^1,2^, Tobias Alfvén^3,4^, Daisuke Yoneoka^5^

^1^Department of Microbiology, Tumor and Cell Biology (MTC), Karolinska Institutet, Sweden

^2^Department of Virology, Tohoku University Graduate School of Medicine, Japan

^3^Department of Global Public Health, Karolinska Institutet, Sweden

^4^Sachs’ Children and Youth Hospital, Sweden

^5^Center for Surveillance, Immunization, and Epidemiologic Research, National Institute of Infectious Diseases, Japan

*Table of Contents*

[Instructions for the shiny application 2](#_Toc199547007)

[Supplementary Tables and Figures 5](#_Toc199547019)

[Supplementary Table 1 5](#_Toc199547020)

[Supplementary Table 2 5](#_Toc199547021)

[Supplementary Figure 1 6](#_Toc199547022)

[Supplementary Figure 2 7](#_Toc199547023)

[Supplementary Figure 3 8](#_Toc199547024)

[Supplementary Figure 4 9](#_Toc199547025)

# **Instructions for the shiny application**

To run the shiny application locally on your computer, follow these steps:

0. Install R and RStudio:

If you have not already installed R and RStudio, download and install them.

• **R**: <https://cran.r-project.org/>

• **RStudio**: <https://posit.co/download/rstudio-desktop/>

1. Download the shiny app files:

You can download all the necessary files from here (<https://github.com/KoKYura/TND_power>).

2. Install required R packages:

Open Rstudio and install the necessary packages by running the following commands in the R console.

Install.packages(“shiny”)

Install.packages(“shinydashboard”)

Install.packages(“DT”)

Install.packages(“JuliaCall”)

3. Run the application:

Open the “shiny/app.R” in Rstudio, and run the application either clicking the “Run App” button in Rstudio or running the following command in the R console:

shiny::runApp()

After running the command, you should see the following page.


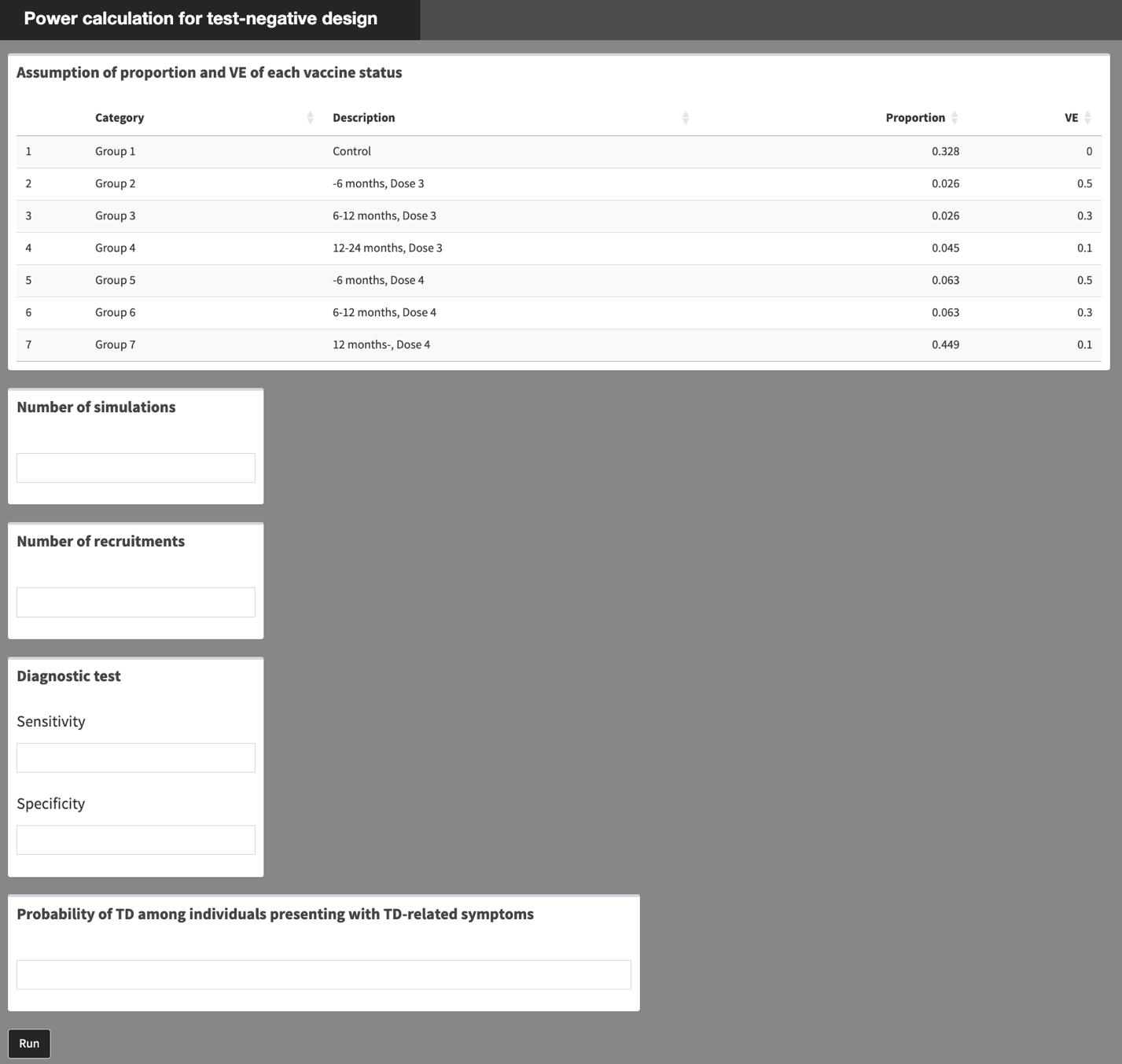


4. Change parameters and run simulations:

Based on the assumptions for your target diseases and study area, modify the following parameters:

- Proportion and vaccine effectiveness (VE) for each vaccination status
- Number of simulations
- Total number of recruitments
- Diagnostic test sensitivity and specificity
- Probability of being diagnosed as target disease (TD)

After setting all parameters, click “Run” to start the simulation. Once the simulation is complete, you will obtain the following plots.


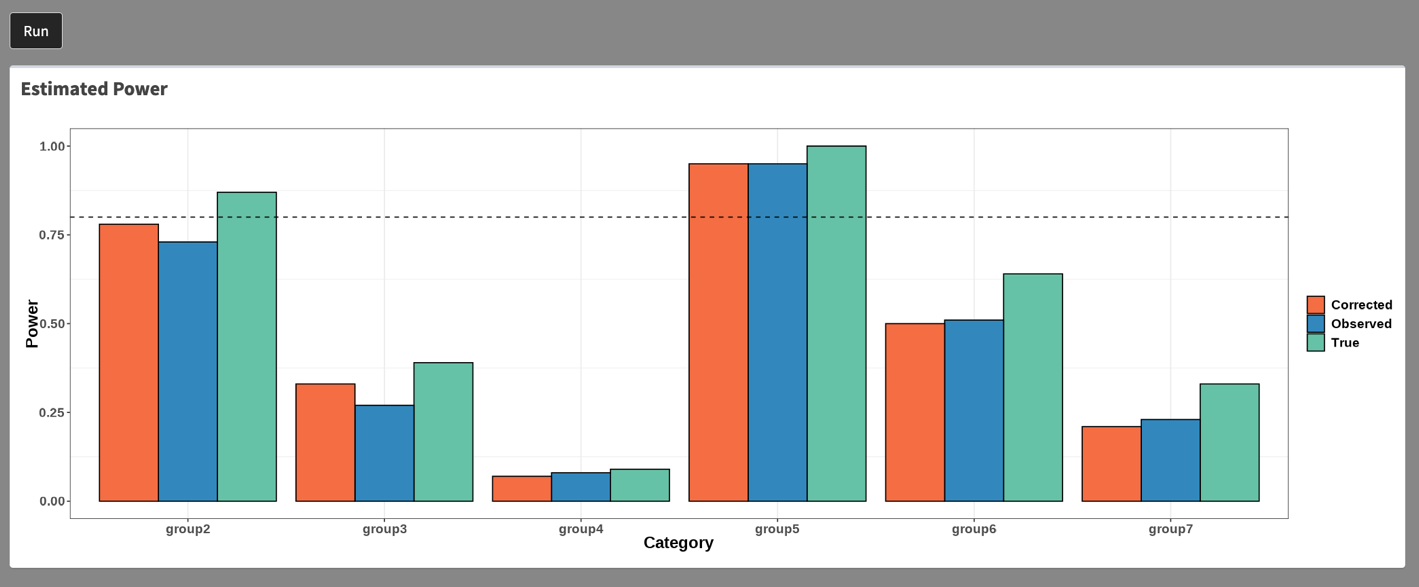


# **Supplementary Tables and Figures**

**Supplementary Table 1**: The alpha error when the bias correction cunducted for an imperfect test with a sensitivity of 60% and a specificity of 98% in each sample size for each vaccination status

| N | –6 months, Dose 3 | 6–12 months, Dose 3 | 12–24 months, Dose 3 | –6 months, Dose 4 | 6–12 months, Dose 4 | 12– months, Dose 4 |
| --- | --- | --- | --- | --- | --- | --- |
| 5000 | 0.029 | 0.025 | 0.022 | 0.028 | 0.011 | 0.023 |
| 6000 | 0.025 | 0.036 | 0.024 | 0.021 | 0.022 | 0.026 |
| 7000 | 0.029 | 0.039 | 0.032 | 0.024 | 0.028 | 0.031 |
| 8000 | 0.031 | 0.024 | 0.025 | 0.017 | 0.027 | 0.025 |
| 9000 | 0.021 | 0.024 | 0.028 | 0.023 | 0.037 | 0.022 |
| 10000 | 0.020 | 0.028 | 0.029 | 0.021 | 0.021 | 0.027 |

**Supplementary Table 2**: The alpha error when the bias correction cunducted for an imperfect test with a sensitivity of 95% and a specificity of 98% in each sample size for each vaccination status

| N | –6 months, Dose 3 | 6–12 months, Dose 3 | 12–24 months, Dose 3 | –6 months, Dose 4 | 6–12 months, Dose 4 | 12– months, Dose 4 |
| --- | --- | --- | --- | --- | --- | --- |
| 5000 | 0.027 | 0.023 | 0.029 | 0.029 | 0.03 | 0.022 |
| 6000 | 0.022 | 0.028 | 0.031 | 0.029 | 0.038 | 0.031 |
| 7000 | 0.027 | 0.025 | 0.035 | 0.029 | 0.027 | 0.034 |
| 8000 | 0.03 | 0.032 | 0.026 | 0.029 | 0.028 | 0.022 |
| 9000 | 0.038 | 0.034 | 0.03 | 0.025 | 0.034 | 0.025 |
| 10000 | 0.033 | 0.033 | 0.031 | 0.025 | 0.04 | 0.03 |

**Supplementary Figure 1**: Estimated true, observed, and bias-corrected vaccine effectiveness (VE) under an imperfect diagnostic test with 60% sensitivity and 98% specificity. Estimates are presented with 95% coverage intervals based on 1,000 Monte Carlo simulations, stratified by vaccination status group and sample size.

**Supplementary Figure 2**: Estimated true, observed, and bias-corrected vaccine effectiveness (VE) under an imperfect diagnostic test with 95% sensitivity and 98% specificity. Estimates are presented with 95% coverage intervals based on 1,000 Monte Carlo simulations, stratified by vaccination status group and sample size.

**Supplementary Figure 3**: Estimated statistical power of true vaccine effectiveness (VE) using a perfect diagnostic test, with VE varying from 60% to 98%. The target vaccination group corresponds to individuals who received Dose 3 of the malaria vaccine within the previous 6 months, with a coverage of 2.6% in the total sample. **The figure illustrates that extremely high VE (>90%) resulted in lower statistical power compared to lower VE levels.**

**Supplementary Figure 4**: Estimated statistical power to detect vaccine effectiveness (VE) for true, observed, and bias-corrected estimates under imperfect diagnostic tests with 80% sensitivity. Two senarios are shown (left) Low probability of being diagnosed as target disease (P(TD) = 5%), and (right) low diagnostic specificity (80%). Results are based on 1,000 Monte Carlo simulations for each vaccination status group and sample size.
